# Bilateral renal and bladder agenesis with exceptional survival without prenatal intervention: a case report

**DOI:** 10.3389/fped.2026.1794522

**Published:** 2026-04-23

**Authors:** M. Heyne-Pietschmann, J. Caccia, S. Tschumi, M. Zeino

**Affiliations:** 1 Pediatric Urology, Department of Pediatric Surgery, Children’s Hospital, University of Bern, Bern, Switzerland; 2 Pediatric Nephrology, Children’s Hospital, University of Bern, Bern, Switzerland

**Keywords:** amnioinfusion, bilateral renal agenesis, dialysis, kidney transplantation, survival

## Abstract

Bilateral renal agenesis (BRA) is typically considered a lethal condition due to severe pulmonary hypoplasia caused by early-onset anhydramnios. We report the case of a male infant who survived without prenatal intervention, underwent complex postnatal nephrological and surgical management, and ultimately received a successful renal transplant. This case highlights the exceptional possibility of survival of a patient with BRA and underscores the importance of multidisciplinary care.

## Introduction

Bilateral renal agenesis (BRA) is a very rare congenital anomaly caused by early disruption of metanephric kidney development, resulting in failure of nephron formation and complete absence of renal tissue. It is most commonly associated with pathogenic variants in key developmental genes and, less frequently with chromosomal aberrations or multifactorial etiologies (1–4). A male predominance has been described (3, 5), and potential embryologic stressors as well as familial occurrence have been reported in the literature (5).

Possible prenatal management strategies include serial amnioinfusions (6–8). Postnatally, immediate cardiopulmonary stabilization is critical, followed by early initiation of renal replacement therapy, most commonly peritoneal dialysis, although hemodialysis may be considered as an alternative modality (9, 10).

BRA is generally regarded as incompatible with life due to severe pulmonary hypoplasia secondary to early-onset anhydramnios (4, 11, 12). Reported survival has been linked to serial prenatal amnioinfusions, but even in such cases, morbidity and mortality remain very high, with reported rates up to 80%–100% (6–8, 13–15). We present a unique case of survival without antenatal intervention, with successful long-term outcome after complex neonatal management.

## Case description

**Figure 1:**
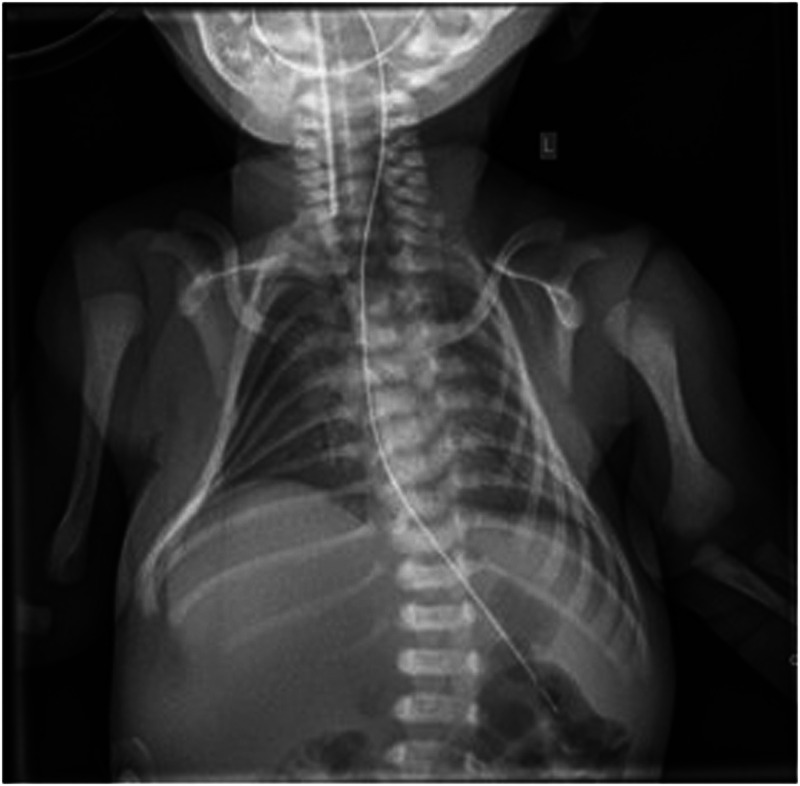
Chest x-ray on first day of life showing severe pulmonary hypoplasia with bell-shaped thorax.

The patient was conceived by intracytoplasmic sperm injection (ICSI) in a dizygotic twin pregnancy, with intrauterine demise of the twin at 8–9 weeks of gestation. At 20 weeks, ultrasound revealed anhydramnios and non-visualization of both kidneys. Genetic testing and serial amnioinfusions were discussed with the parents but declined. Delivery was performed by secondary cesarean section at 36 + 2 weeks of gestation. The male infant was born with a birth weight of 1,765 g and APGAR scores of 2, 3 and 4. He required immediate intubation, high-frequency oscillatory ventilation, surfactant administration, and inhaled nitric oxide for severe pulmonary hypoplasia and severe pulmonary hypertension (Figure 1). In echocardiography, a dysplastic pulmonary valve with mild stenosis, a ventricular septum defect (VSD) and a small patent ductus arteriosus (PDA) with a left-to-right shunt were also observed. Clinical features included Potter facies with a retrognathia, narrow forehead and low-set ears. Ultrasound confirmed bilateral renal agenesis and bladder agenesis. As in the prenatal period, no postnatal genetic testing was performed at the parents’ request.

A peritoneal dialysis (PD) catheter was inserted within the first 24 h of life, and peritoneal dialysis was initiated after initial stabilization on the third day of life. Due to a pneumothorax on the left side, he required chest tube insertion on the second day of life. The patient gradually improved and was extubated on day 10 of life. In the neonatal period, he underwent bilateral herniotomy and retrograde urethrography, which suggested absence of the bladder with canopy of prostatic urethra with normal appearing urethra. Postoperatively, he developed necrotizing enterocolitis, which was treated conservatively. He was discharged home at two months of age on peritoneal dialysis. The following years were challenging, marked by recurrent respiratory infections and deterioration of lung function with persistence of pulmonary arterial hypertension with necessity to start sildenafil and bosentan, together with home respiratory support (nighttime intermittent high flow therapy and oxygen via a nasal cannula as needed). He was diagnosed with type I laryngomalacia, which was treated with laser supraglottoplasty. In addition, he experienced complications related to peritoneal dialysis, including catheter dysfunction and episodes of PD-associated peritonitis, necessitating close, interdisciplinary management. We had to switch dialysis modality to chronic intermittent hemodialysis at age 26 months due to ultrafiltration failure and volume overload with worsening of the pulmonary situation and decompensation of pulmonary hypertension. He required invasive mechanical ventilation for 12 days, including adjunctive inhaled nitric oxide therapy. He subsequently remained clinically stable under hemodialysis but suffered multiple catheter-associated complications (tunnel infection, catheter-associated bacteremia, catheter leakage, dysfunction) and needed 4 hemodialysis-catheter changes.

**Figure 2:**
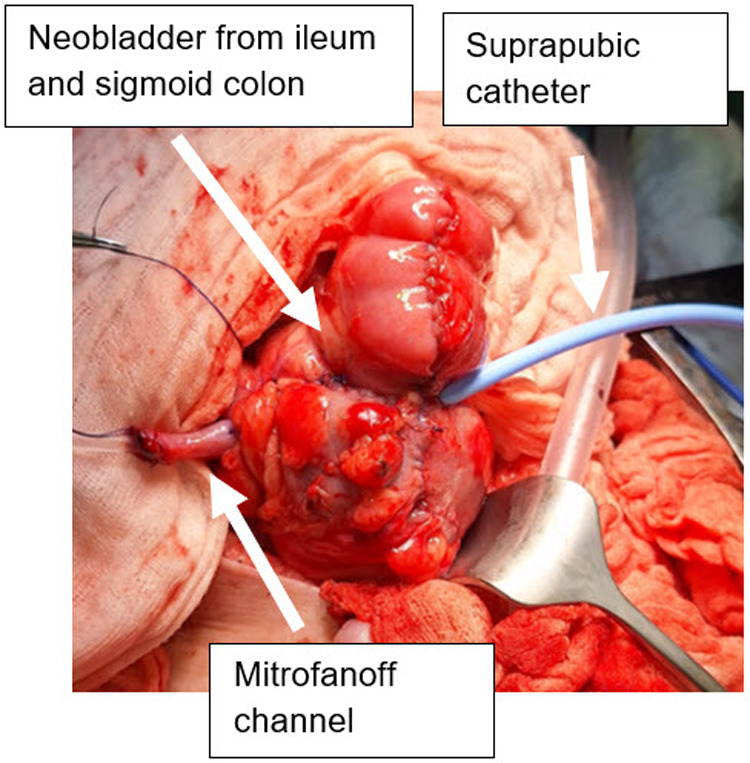
Intraoperative status after construction of the neobladder from ileum and sigmoid colon and mitrofanoff channel.

**Figure 3:**
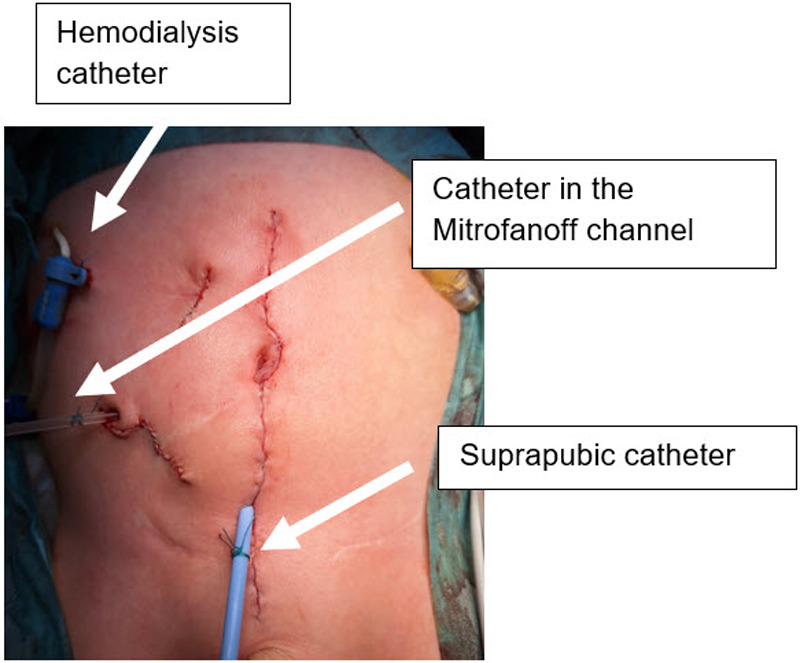
Postoperative status with Mitrofanoff stoma located in the right lower quadrant.

At the age of three years, he underwent laparotomy with extensive adhesiolysis, creation of a continent neobladder from ileum and sigmoid colon with a Mitrofanoff channel in the right lower abdomen in VQZ technique and orchiectomy for an intra-abdominal atrophic testis in one stage (Figures 2, 3). Intraoperatively, non-rotation of the intestine was observed, and complete absence of kidneys and ureters, with only a small overlying prostatic urethra were confirmed. Postoperatively, a catheter was left in the Mitrofanoff channel for three weeks to serve as a stent, and an additional suprapubic catheter was placed for urinary drainage. Three weeks after surgery, contrast imaging of the neobladder was performed to exclude urinary leakage. Subsequently, clean intermittent catheterization (CIC) with irrigation of the neobladder using 50 mL of saline solution twice daily was initiated. The postoperative course was uneventful, with no complications and a rapid recovery.

**Figure 4:**
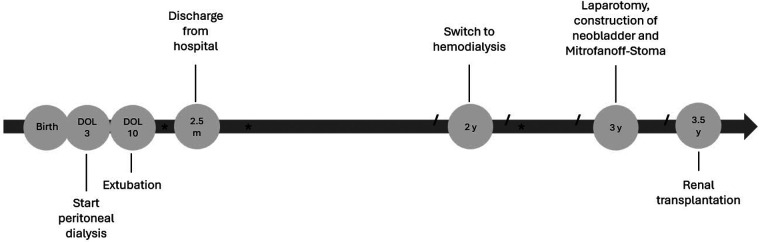
Course from birth to renal transplantation. *, infection of peritoneal dialysis/hemodialysis catheter; **/**, change of hemodialysis catheter; DOL, day of life; m, months, y, years.

Six months later, he received living donor kidney transplantation from a well-matched related donor. Due to the extensive adhesiolysis required and the limited intra-abdominal space, primary abdominal closure was not feasible. Therefore, a mesh in combination with a vacuum-assisted closure (VAC) system was applied. Secondary closure of the abdomen was successfully performed one week later. Postoperatively, he developed a large lymphocele, which was successfully drained percutaneously. Graft function has been excellent since transplantation. Clean intermittent catheterization via the Mitrofanoff channel is performed without difficulty. Regarding his pulmonary status, the patient has no longer required nocturnal flow therapy or home oxygen since transplant. Drug therapy with sildenafil and bosentan has been continued. Neurologically, he shows a global development delay. However, he is achieving milestones, walking independently and speaking several words and his developmental progress has accelerated since transplantation. The clinical course from birth to renal transplantation is summarized in Figure 4.

## Discussion

BRA has traditionally been regarded as uniformly lethal due to the development of severe pulmonary hypoplasia secondary to early-onset anhydramnios. Most published reports describe early neonatal death, with survival largely limited to cases where serial amnioinfusions were performed. Even in these cases, survival beyond the neonatal period is rare. Those infants who survive the immediate postnatal period typically also face substantial morbidity and mortality in the months and years preceding transplantation, often due to complications associated with dialysis, severe infections, or other significant concomitant malformations (6–8, 13–15).

A review of the literature shows only a handful of patients who have survived long enough to receive renal transplantation. Bienstock et al., Sheldon et al. and Riddle et al. collectively reported 17 cases of BRA treated with serial amnioinfusions, but only two of these patients survived to receive a renal transplant at the ages of 2.5 years and 20 months (6, 7, 13). George et al. described a child with BRA and duodenal atresia with normal pulmonary function, diagnosed postnatally, who survived four years and remained on dialysis while awaiting transplantation. The unusually favorable pulmonary outcome in this case may have been facilitated by the presence of gastrointestinal atresia, which could have contributed to maintaining higher amniotic fluid levels in utero (14). In addition, several reports of monoamniotic twin pregnancies discordant for BRA demonstrated the high mortality with death of the affected twin within the first two months of life (16–19). More recently, the RAFT Trial (Renal Anhydramnios Fetal Therapy Trial) has provided systematic evidence for the role of serial amnioinfusions. In this prospective, multicenter study, 82% of infants, who received prenatal amnioinfusions, survived at least two weeks, but only 35% survived to hospital discharge. Importantly, no infants survived without antenatal intervention. Enrollment was stopped early due to the high morbidity and mortality observed as well as the observed efficacy with regard to the primary outcome (2 week survival and dialysis access placement) (8).

Our patient represents a rare exception, demonstrating survival without prenatal therapy. The etiology of BRA in our patient is unknown as parents declined genetic testing, the mother had no known risk factors. To our knowledge, no similar case has been reported. The reason for this child's survival remains unknown; despite complete anhydramnios, he may have achieved just enough pulmonary development for maximal postnatal cardiopulmonary support to be effective. This might have allowed the comprehensive postnatal management with intensive interdisciplinary neonatal care, early peritoneal dialysis, staged urological reconstruction, and successful kidney transplantation to ultimately result in survival. This directly challenges the traditional perception of bilateral renal agenesis as a uniformly lethal condition and demonstrates that multidisciplinary perinatal and postnatal care can lead to survival in very rare cases. It underlines the therapeutic possibilities given to families of infants with BRA. Nevertheless, these children face many medical and surgical challenges. At the same time, the exceptional nature of this case emphasizes the need for realistic counseling and also raises an ethical dilemma: even with interventions, overall morbidity and mortality remain extremely high.

## Conclusion

This case demonstrates extraordinary survival in an infant with bilateral renal and bladder agenesis without antenatal intervention, his survival remains truly remarkable. Intensive neonatal and nephrological care, early dialysis, staged reconstruction, and kidney transplantation enabled long-term survival and favorable development, challenging the traditional perception of BRA as universally lethal and highlighting the importance of individualized, multidisciplinary care.

## Data Availability

The original contributions presented in the study are included in the article/Supplementary Material, further inquiries can be directed to the corresponding author.
